# It's not all in the brain

**DOI:** 10.7554/eLife.30561

**Published:** 2017-08-29

**Authors:** Lauren J Francey, John B Hogenesch

**Affiliations:** 1 Divisions of Human Genetics and ImmunobiologyCincinnati Children's Hospital Medical CenterCincinnatiUnited States

**Keywords:** bmal1, sleep, skeletal muscle, homeostasis, arntl, physiology, Mouse

## Abstract

A clock gene expressed in skeletal muscle plays a bigger role in regulating sleep than it does in the brain.

**Related research article** Ehlen JC, Brager AJ, Baggs J, Pinckney L, Gray CL, DeBruyne JP, Esser KA, Takahashi JS, Paul KN. 2017. *Bmal1* function in skeletal muscle regulates sleep. *eLife* **6**:e26557. doi: 10.7554/eLife.26557

“Great workout, I’m going to sleep like a baby tonight!” Ever wondered why strenuous exercise often leads to a great night’s sleep? A new study in eLife could help researchers explain this (Ehlen et al., 2017).

All mammals need to sleep, but why and how we sleep still remains largely a mystery. According to the ‘two-process model’, first proposed 35 years ago, the sleep-wake cycle is a product of two distinct mechanisms: the sleep homeostat that governs how much sleep you need, and the circadian clock that dictates when you get it (Borbély, 1982).

Sleep is regulated by different regions in the brain and by a number of different chemical messengers and genes. In the last three decades, circadian clock genes were identified that regulate sleep timing (for a recent review, see (Takahashi, 2017)). Experiments in humans and animal models revealed that, far from being distinct, many core clock genes, for example,* Clock* or *Npas2*, also regulate sleep homeostasis (Laposky et al., 2005; Franken et al., 2006; Viola et al., 2007; Allebrandt et al., 2010; Zhou et al., 2014; Mang et al., 2016). Further, some of the genes that regulate the sleep homeostat, such as *Dec2*, also control the expression of clock genes (He et al., 2009; Pellegrino et al., 2014).

The *Bmal1* gene is the only clock gene required for circadian rhythms in mammals. Under conditions of constant darkness – how circadian rhythms are typically studied – mice that completely lacked *Bmal1* lost their 24-hour rhythms and slept longer than mice that still had the gene. These mice also responded differently to sleep deprivation (Laposky et al., 2005). The standard response to sleep deprivation is to sleep longer and more deeply. However, silencing *Bmal1* throughout the brain and body impaired the ability of the mice to rebound from sleep deprivation. Conventional wisdom suggests that *Bmal1* exerts its influence in the brain. Indeed, when *Bmal1* was selectively deleted in histaminergic neurons, the mice had fragmented sleep and didn’t recover from sleep deprivation as well (Yu et al., 2014).

Now in eLife, Ketema Paul and colleagues – including Christopher Ehlen and Allison Brager as co-first authors – report that conventional wisdom is wrong (Ehlen et al., 2017). Ehlen et al. decided to test whether restoring the expression of the *Bmal1* gene selectively in the brain of *Bmal1-*deficient mice would rescue their response to sleep deprivation: it didn’t. However, restoring *Bmal1* in skeletal muscle did. Mice with *Bmal1* expressed in the skeletal muscle slept normally, whereas mice with *Bmal1* expressed in the brain slept abnormally.

Moreover, when the researchers selectively knocked-out *Bmal1* in the skeletal muscle, the mice couldn’t recover from sleep deprivation as well, similar to mice that completely lacked the gene. Conversely, when *Bmal1* was over-expressed in skeletal muscle, it made them resistant to even longer periods of sleep deprivation.

Collectively, these results show that the *Bmal1* gene in the skeletal muscle regulates responses to sleep deprivation. Not only is the two-process model obsolete – the dogma that sleep is governed solely by the brain has been upended. However, important questions remain. For example, does *Bmal1* in the skeletal muscle regulate the response to sleep deprivation on its own, or are its partners *Clock* and *Npas2* also involved? What specific signals does the skeletal muscle send to the brain to trigger the onset of sleep and how are they conveyed? Could timed exercise be used to amp up *Bmal1* expression and lead to better sleep?

For decades, we’ve known that the body influences sleep. Think about how you felt the last time you had ‘food coma’, how tired you feel when you are sick or after a day in the sun, or how well you sleep after a great workout (provided sore muscles don't keep you awake all night as it does me -- JBH). For the first time, Ehlen et al. – who are based at Morehouse School of Medicine, the University of Florida, the Walter Reed Army Institute of Research, the University of Texas Southwestern Medical Center, and the University of California Los Angeles – show that a gene outside of the brain controls how mammals rebound from sleep deprivation. These findings may open new avenues for treating sleep disorders, potentially through exercise. “Eat well, exercise, and get lots of sleep” may be old advice, but now it’s supported by modern genetics.
